# Room-temperature crystallography using a microfluidic protein crystal array device and its application to protein–ligand complex structure analysis†

**DOI:** 10.1039/d0sc02117b

**Published:** 2020-08-25

**Authors:** Masatoshi Maeki, Sho Ito, Reo Takeda, Go Ueno, Akihiko Ishida, Hirofumi Tani, Masaki Yamamoto, Manabu Tokeshi

**Affiliations:** Division of Applied Chemistry, Faculty of Engineering, Hokkaido University Kita 13 Nishi 8, Kita-ku Sapporo 060-8628 Japan m.maeki@eng.hokudai.ac.jp tokeshi@eng.hokudai.ac.jp +81-11-706-6745 +81-11-706-6745 +81-11-706-6744; RIKEN SPring-8 Center 1-1-1 Kouto, Sayo-cho Sayo-gun Hyogo 679-5148 Japan; Graduate School of Life Science, University of Hyogo 3-2-1 Kouto, Kamigori Ako Hyogo 678-1297 Japan; ROD (Single Crystal Analysis) Group, Application Laboratories, Rigaku Corporation 3-9-12 Matubara-cho Akishima Tokyo 196-8666 Japan; Graduate School of Chemical Sciences and Engineering, Hokkaido University Kita 13 Nishi 8, Kita-ku Sapporo 060-8628 Japan

## Abstract

Room-temperature (RT) protein crystallography provides significant information to elucidate protein function under physiological conditions. In particular, contrary to typical binding assays, X-ray crystal structure analysis of a protein–ligand complex can determine the three-dimensional (3D) configuration of its binding site. This allows the development of effective drugs by structure-based and fragment-based (FBDD) drug design. However, RT crystallography and RT crystallography-based protein–ligand complex analyses require the preparation and measurement of numerous crystals to avoid the X-ray radiation damage. Thus, for the application of RT crystallography to protein–ligand complex analysis, the simultaneous preparation of protein–ligand complex crystals and sequential X-ray diffraction measurement remain challenging. Here, we report an RT crystallography technique using a microfluidic protein crystal array device for protein–ligand complex structure analysis. We demonstrate the microfluidic sorting of protein crystals into microwells without any complicated procedures and apparatus, whereby the sorted protein crystals are fixed into microwells and sequentially measured to collect X-ray diffraction data. This is followed by automatic data processing to calculate the 3D protein structure. The microfluidic device allows the high-throughput preparation of the protein–ligand complex solely by the replacement of the microchannel content with the required ligand solution. We determined eight trypsin–ligand complex structures for the proof of concept experiment and found differences in the ligand coordination of the corresponding RT and conventional cryogenic structures. This methodology can be applied to easily obtain more natural structures. Moreover, drug development by FBDD could be more effective using the proposed methodology.

## Abstract 2

Room temperature protein crystallography and its application to protein–ligand complex structure analysis was demonstrated using a microfluidic protein crystal array device.

## Graphical abstract

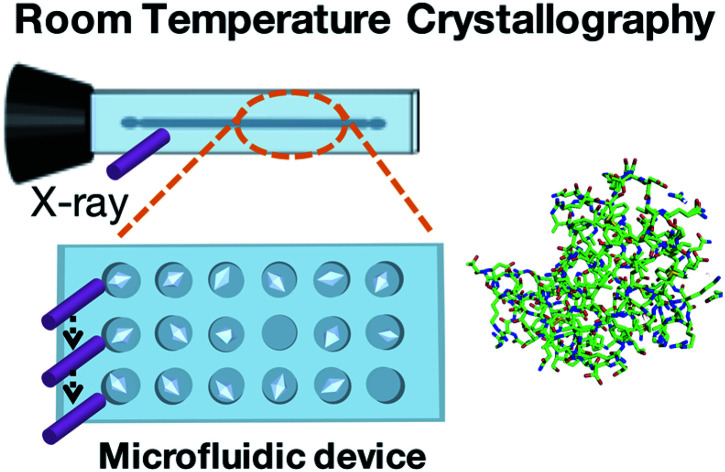

## Introduction

The three-dimensional (3D) structure of a protein–ligand complex provides significant information to elucidate protein functions and can be applied to drug discovery.^1,2^ In particular, structure-based (SBDD) and fragment-based (FBDD) drug design strategies accelerate the development of novel drugs. These methodologies provide indispensable information for the *in silico* screening of ligand fragments.^3,4^ Thus, the combination of SBDD or FBDD with artificial intelligence can lead to cost-effective and high-throughput drug development in the future.

High-throughput ligand screening based on protein crystallography is still challenging in the fields of pharmaceutical sciences and structural biology. Generally, diffraction data collection for protein crystal structure analysis is carried out at cryogenic temperature to avoid X-ray radiation damage. Therefore, protein crystallography-based ligand screening requires complicated and laborious procedures for several thousands of candidate compounds and fragments. These processes include protein crystallization, soaking of the protein crystal into a ligand solution, soaking of the protein–ligand complex into a cryoprotectant, freezing, setting up of the X-ray diffractometer, and X-ray irradiation. Previously, these procedures were manually handled using a cryo-loop for each crystal. However, recently, automation of the 3D protein structure analysis process, which includes automated sample changers as well as fully automated data processing, has been developed to accelerate the structure determination process. Nevertheless, the lack of a system or interface to connect the high-throughput preparation process of the protein–ligand complex to automated measurement and data processing is the bottleneck of the application of protein crystallography-based ligand screening to SBDD and FBDD.

From the structural biology and crystallography viewpoints, protein structure determination under cryogenic conditions has significantly contributed towards the understanding of protein structures and functions. However, freezing of the protein crystals affects their structures in several ways, such as restricting the side-chain conformation and masking secondary binding sites, which contribute to allosteric regulation.^5–9^ On the other hand, room-temperature (RT) protein crystallography allows the elucidation of the 3D structure in an environment that approaches physiological conditions. In addition, RT protein crystallography should provide better understanding of the interactions between the target proteins and ligands, leading to more detailed compound structural data. The major difference between cryo-crystallography and RT protein crystallography (*e.g.*, serial femtosecond crystallography)^10–12^ is that in the latter technique, a number of protein crystals need to be measured to determine the 3D structure. Because the X-ray radiation damage is more serious in the RT technique, small wedges of the diffraction data are collected from each crystal and merged to determine the 3D structure. Thus, for RT protein crystallography characterization, numerous preparations and measurements of the protein–ligand complex crystals are required and the automation methodology from sample preparation to data processing is more significant than that of the conventional cryo-crystallography technique. RT protein crystallography offers attractive advantages for structural biology and drug discovery; however, a system or device that can be applied to the automation of RT protein crystallography, including ligand or fragment screening, is yet to be developed.

Microfluidic platforms are highly desirable technologies to develop such an automated system and enable high-throughput protein crystallization condition screening,^13–18^ low sample consumption,^19^ control of protein crystal growth,^20–23^*in situ* X-ray diffraction measurement,^24–28^ and other applications.^29–31^ A variety of microfluidic devices have been reported for conventional protein crystallography as well as for their application to serial femtosecond crystallography using X-ray-free electron laser technology. Moreno-Chicano *et al.* reported a fixed target device for high-throughput protein–ligand structure determination applicable for serial femtosecond crystallography.^32^ In the serial femtosecond crystallography experiment, because one still image can be taken from a crystal, a large number of crystals are required. In contrast, with a synchrotron light source routinely utilized for protein crystallography, a continuous image can be taken from a crystal. Therefore, it is desirable to develop a dedicated device which can easily utilize in synchrotron using microfluidics to reduce sample consumption. Although RT protein crystallography was demonstrated using microfluidics such as cell trap devices,^33^ microwells,^34,35^ and microdroplets,^36^ protein–ligand complex 3D structure analysis has not been well investigated to date due to the lack of a device providing the high-throughput preparation of numerous the protein–ligand complex crystals and automated measurement systems. For application to RT protein–ligand complex crystallography, the simultaneous preparation of protein–ligand complex crystals prior to X-ray diffraction and sequential X-ray diffraction measurements is a critical requirement, while a user-friendly interface is also desirable for novice crystallographers such as biologists and pharmacologists.

**Fig. 1:**
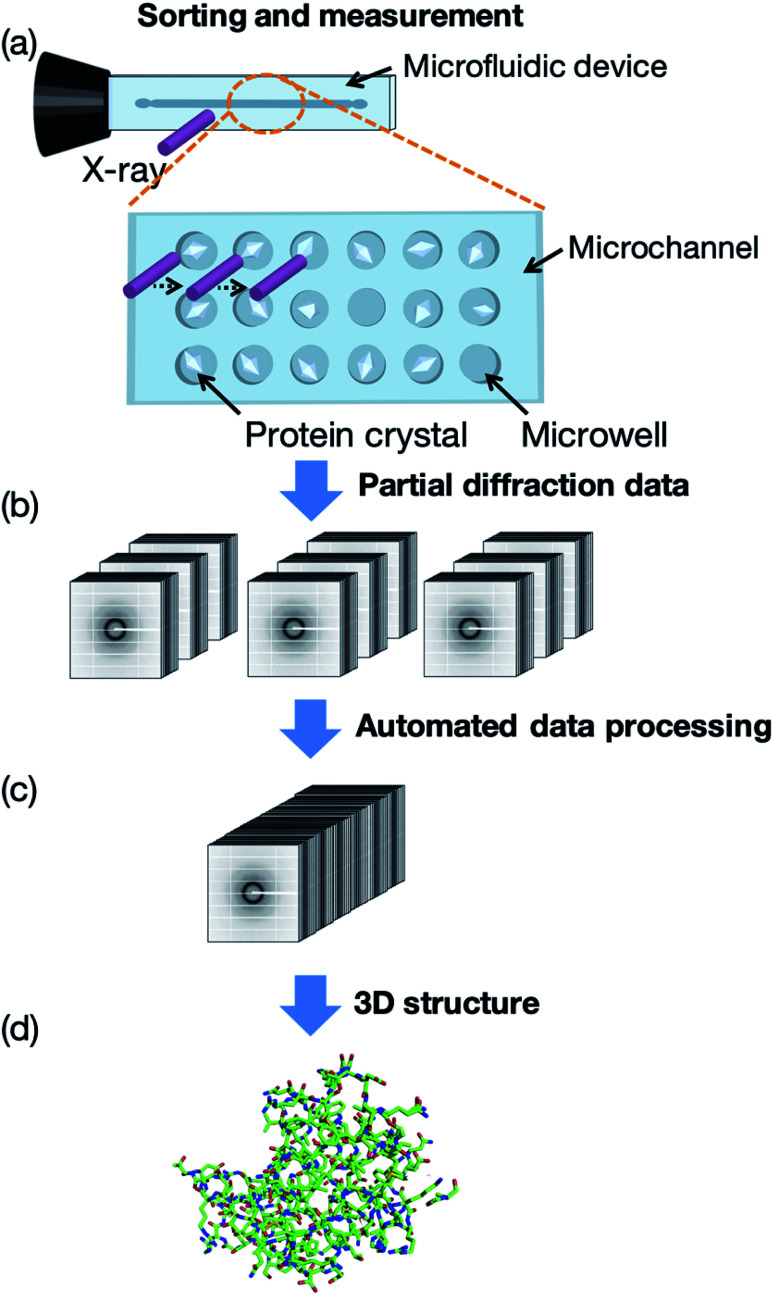
Concept of room-temperature (RT) protein crystallography using the proposed microfluidic protein crystal array device. (a) Protein crystals are sorted into microwells and sequentially measured to collect partial X-ray diffraction data. (b and c) Partial diffraction data are automatically processed and merged to obtain a complete diffraction data set. (d) Three-dimensional (3D) protein structure is calculated from the data set.

In this study, we first developed a microfluidic device for RT protein crystallography, in particular semi-automated protein–ligand complex 3D structure analysis. Our measurement concept, named the “protein crystal array,” is presented in Fig. 1. In this concept, protein crystals are fixed into microwells within the microfluidic device. A few microliters of protein crystal suspension is introduced by a micropipette and subsequent measurement is attained by exposing each protein crystal to X-ray. We determined 3D protein–ligand complex structures from the multiple protein crystals sorted into the microwells with subsequent automated diffraction data processing. Ligand screening for trypsin using eight model compounds was demonstrated for the proof of concept experiment. The proposed method provided protein–ligand complex information on the compound structure, binding site, significant functional groups, and hydrated water molecules based on the 3D structures.

## Experimental section

### Materials

Thaumatin from *Thaumatococus daniellii*, trypsin from bovine pancreatic, sodium chloride, sodium acetate, potassium sodium tartrate, acetic acid, acetone, and 2-propanol were purchased from Fujifilm Wako Pure Chemical Corporation (Osaka, Japan). Lysozyme chloride from egg white was purchased from Nacalai Tesque (Kyoto, Japan). Selenourea and trichloro(1*H*,1*H*,2*H*,2*H*-perfluorooctyl)silane were purchased from Sigma-Aldrich (St. Louis, MO, USA). *N*-(2-Acetamido)iminodiacetic acid (ADA) and 4-(2-hydroxyethyl)-1-piperazineethanesulfonic acid (HEPES) were obtained from Dojindo laboratories (Kumamoto, Japan). *p*-Toluenesulfonic acid monohydrate and all ligands for trypsin were purchased from Tokyo Chemical Industry Co., Ltd. (Tokyo, Japan). Polydimethylsiloxane (PDMS; SILPOT 184 W/C) was purchased from Dow Corning Toray Co., Ltd. (Tokyo, Japan), while SU-8 3050 and the SU-8 developer were purchased from Nippon Kayaku Co., Ltd (Tokyo, Japan). Silicon wafers were obtained from Global Top Chemical (Tokyo, Japan) and cyclic olefin polymer (COP) films, 40 μm in thickness, were purchased from Zeon (Tokyo, Japan).

### Fabrication of the microfluidic devices

**Fig. 2:**
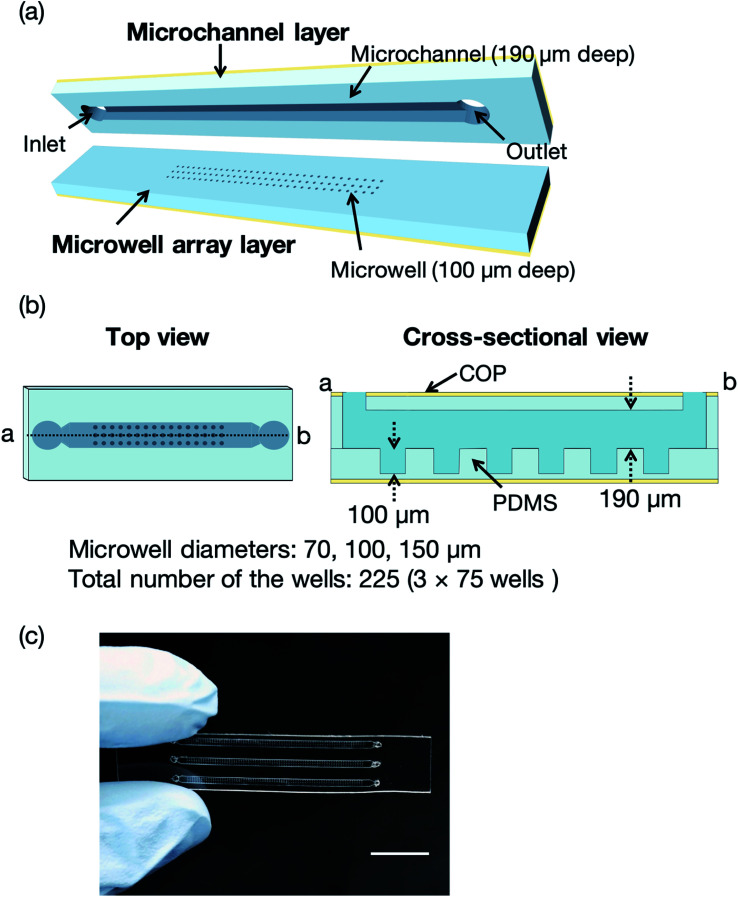
(a) Three-dimensional (3D) perspective view of the microfluidic device comprising microchannel and microwell array layers with depths of 190 and 100 μm, respectively. (b) Top and cross-sectional views (microwell diameters = 70, 100, and 150 μm) and (c) photograph of the microfluidic device. The total number of the wells was 225 (3 × 75 wells). The scale bar represents 1 cm. Definitions: PDMS, polydimethylsiloxane; COP, cyclic olefin polymer.

Fig. 2 illustrates a microfluidic device for the proof of concept experiments composed of a thin microchannel layer and a microwell array layer. To reduce the background scattering, we selected a thin-layer microfluidic device for the proof of concept experiments. Each layer was fabricated by a standard soft lithography process.^37^ In brief, SU-8 3050 was poured onto silicon wafers and washed with acetone and 2-propanol. The silicon wafers were spin-coated (MS-A100, Mikasa Shoji, Tokyo, Japan) to obtain 100 and 190 μm-thick SU-8 layers for the microchannel and microwell array layers, respectively. We fabricated three SU-8 molds for the microwell array layer with microwell diameters of 70, 100, and 150 μm. The total number of the wells was 225 (3 × 75 wells). After the soft baking process, the silicon wafers with photomasks (12 700 dpi, Unno Giken Co., Ltd., Tokyo, Japan) were exposed to ultraviolet (UV) light using a mask aligner (M-1S, Mikasa Shoji). The silicon wafers were baked onto a hotplate to cross-link the SU-8 and subsequently soaked into the SU-8 developer. The SU-8 molds were treated with trichloro(1*H*,1*H*,2*H*,2*H*-perfluorooctyl)silane vapor and PDMS was poured onto the molds to create the microchannel and microwell array layers. The SU-8 molds for these two layers were spin-coated using the spin coater to control the thickness (200 and 120 μm, respectively) of each layer. The PDMS-coated SU-8 molds were then baked on a hotplate and subsequently bonded with COP film by oxygen plasma treatment (CUTE-1MP/R, Femto Science, Gwangju, Korea). Finally, the microchannel and microwell array layers were cut out from the molds and the microchannel was aligned to the microwell array.^21^ To evaluate the mass production feasibility of the microfluidic device for protein–ligand screening, microfluidic devices solely comprising the PDMS microchannel and microwell layers (without a COP layer) were fabricated by a typical soft lithography technique.^37^ Degassed PDMS was poured onto the silicon wafers, which were then baked in an oven for an hour at 80 °C. The microchannel and microwell array layers were then bonded by oxygen plasma treatment. The total thickness of the microfluidic device was <4 mm to reduce background scattering.

### Preparation of the protein crystals and ligand solutions

The purchased proteins were used for the crystallization experiments without further purification. Thaumatin, lysozyme, and trypsin were used as model proteins. The protein concentrations were measured using a NanoDrop™ instrument (ND-ONE-W, Thermo Scientific, Tokyo, Japan). The protein and precipitant solutions were filtered through 0.2 μm syringe filters (Minisart RC4 or RC25, Sartorius Stedim Biotech, Gottingen, Germany). For thaumatin and lysozyme, the protein solutions were mixed with each precipitant solution and stored in an incubator (MIR-154-PJ, Panasonic, Osaka, Japan) at 20 °C. The thaumatin crystal suspension was prepared by mixing 40 mg mL^−1^ thaumatin solution in 0.1 M ADA buffer at pH 6.5 with 1.5 M potassium sodium tartrate in 50 mM HEPES buffer at pH 7.5. The lysozyme crystal suspension was prepared by mixing 50 mg mL^−1^ lysozyme solution in 50 mM acetate buffer at pH 4.6 with 1.0 M sodium chloride and 50 mM acetate buffer at pH 4.5. Trypsin crystals were prepared by the vapor diffusion method. The trypsin was dissolved to 30–60 mg mL^−1^ in a buffer containing 25 mM HEPES at pH 7.0 and 5 mM calcium chloride. Crystals of the apo form were grown at 277 K after a few days with a reservoir solution of 30% polyethylene glycol 3350, 0.2 M lithium sulfate, and 0.1 M Tris–HCl (pH 8.5).

**Fig. 3:**
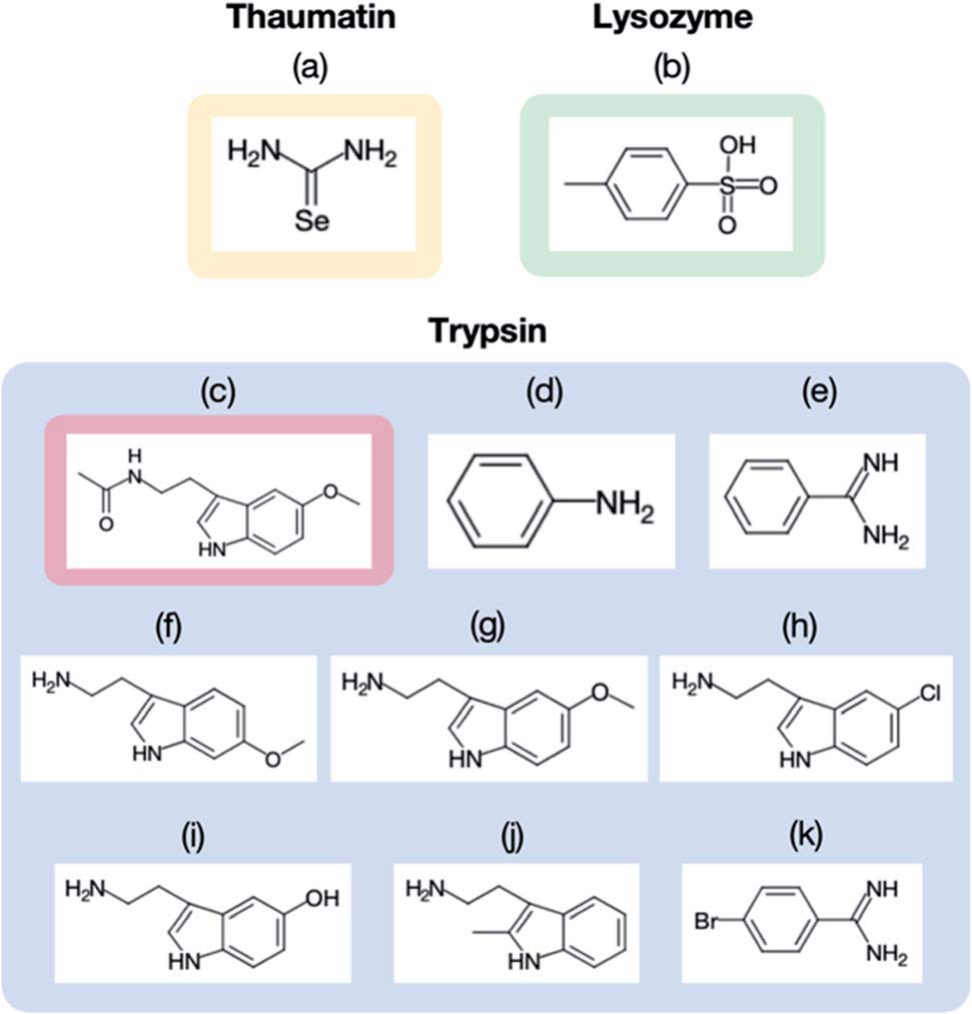
Ligand materials used for (a) thaumatin, (b) lysozyme, and (c–m) trypsin: (a) selenourea, (b) *p*-toluenesulfonic acid, (c) melatonin, (d) aniline, (e) benzamidine, (f) 6-methoxytryptamine, (g) 5-methoxytryptamine, (h) 5-chlorotryptamine, (i) serotonin, (j) 2-methyltryptamine, and (k) 4-bromobenzamine. All the ligands used for trypsin, aside from melatonin (c), were observed in the crystal structures.

Fig. 3 displays the structures of the ligands used in this study for (a) thaumatin, (b) lysozyme, and (c–k) trypsin. We used selenourea and *p*-toluenesulfonic acid as the ligands for thaumatin and lysozyme, respectively. For thaumatin, 100 mM selenourea in 1.5 M potassium sodium tartrate and 50 mM HEPES buffer at pH 7.5 were used as the ligand solution. For lysozyme, *p*-toluenesulfonic acid was dissolved in 1.0 M sodium chloride at pH 4.5 and 0.1 M acetate buffer, and its concentration was adjusted to 300 mM. We also employed nine ligands for trypsin, namely (c) melatonin, (d) aniline, (e) benzamidine, (f) 6-methoxytryptamine, (g) 5-methoxytryptamine, (h) 5-chlorotryptamine, (i) serotonin, (j) 2-methyltryptamine, and (k) 4-bromobenzamine. The hydrophobic ligands (aniline, 6-methyoxytryptamine, 5-methoxytryptamine, 4-bromobenzamidine) were dissolved in a reservoir solution with 10–20% dimethyl sulfoxide (DMSO), while the hydrophilic ligands were dissolved in the reservoir solution. A concentration of 50 mM was used for all ligands aside from aniline (500 mM).

### Microfluidic sorting of the protein crystals and application to ligand screening

**Fig. 4:**
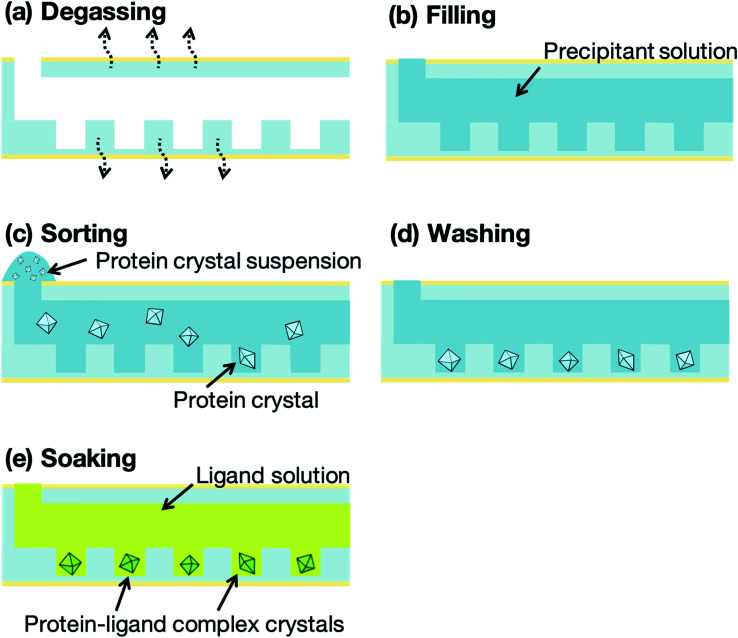
Schematic illustration of the protein crystal sorting and ligand screening experiments: (a) Degassing of the microfluidic device for 10 min. (b) Filling of the microchannel with a precipitant solution using a micropipette. (c) Pipetting of the protein crystal suspension (few microliters) onto the inlet and subsequent pipetting out from the outlet; the protein crystals remain trapped in the microwells. (d) Washing with the precipitant solution to remove the untrapped crystals. (e) Introduction of the ligand solution into the microchannel to prepare the protein–ligand complex crystals simultaneously.

Fig. 4 presents a schematic illustration of the protein crystal sorting and ligand screening experiments. The microfluidic devices were degassed, using a vacuum pump and desiccator, for 10 min prior to the sorting experiment (Fig. 4a). The microfluidic device was then removed from the desiccator and a washing solution, prepared by mixing the buffer solution for the protein with the precipitant solution, was pipetted onto the microfluidic device inlet to fill the microchannel with the solution (Fig. 4b). About 10 μL protein crystal suspension was also pipetted onto the inlet (Fig. 4c). The liquid from the filled microchannel was then pipetted out from the outlet to sort the protein crystals into the microwells. Next, 2 μL of the microchannel content was aspirated through the outlet in 2 s and the collected solution was redropped onto the inlet. This crystal sorting process was repeated fivefold. The untrapped protein crystals were then removed using 10 μL of the washing solution (Fig. 4d). For the preparation of the protein–ligand complexes, the ligand solutions were introduced into the microchannels after the washing step (Fig. 4e). The protein crystals were then soaked into the ligand solution for 5 min to obtain the protein–ligand complex. These procedures were carried out on-site at the synchrotron facilities prior to the X-ray diffraction measurement. To compare the differences in the 3D structures, flash-cooled soaking crystals using liquid nitrogen with additional 20% glycerol as the cryoprotectant were prepared with the same ligand concentrations.

### X-ray diffraction measurement and crystal structure analysis

**Fig. 5:**
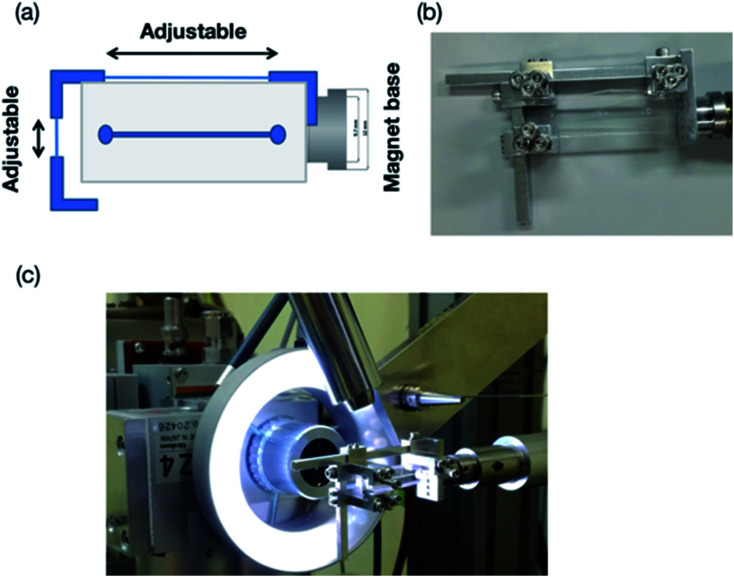
Holding to fix the microfluidic devices for the X-ray diffraction experiment. (a) Schematic illustration and (b) photograph of the holder, onto which the microfluidic device was fixed, which was (c) mounted on a goniometer.

X-ray diffraction data were collected at the beamline BL26B2,^38^ SPring-8 (Japan). The experimental procedures were slightly modified from our previously reported on-device X-ray diffraction measurement procedures.^26^ Both the thin-layer (PDMS–COP) and thick-layer (PDMS only) microfluidic devices were fixed on a goniometer head using a holing tool (Fig. 5). This tool was used to fix the device with screws to prevent it from bending and shifting during the diffraction experiments. Notably, because of radiation damage, a hundredth X-ray dose limitation was imposed on the room-temperature (RT) experiment. This resulted in a shorter period of total exposure, and thus a smaller number of oscillation frames, per crystal compared to that of the cryo-temperature experiment. In this study, three to twenty crystals were used for collecting each complete dataset.

All the diffraction experiments were performed using Beamline Scheduling Software (BSS),^39^ the data collection graphical user interface (GUI) at the SPring-8 PX beamlines. The irradiation points on each crystal were manually determined, by identification from the digital microscope image, and then registered to a list in the BSS GUI. Once all the irradiation points were specified, the diffraction experiment was performed automatically by BSS. The diffraction data were measured at a wavelength of 1.0 Å, aside from 4-bromobenzamidine (0.918 Å), with 0.5 s exposure time and a 0.5° oscillation step at RT. The X-ray beam size was 100 μm (full width at half maximum) and Gaussian-shape, and the photon flux was 1.2 × 10^11^ photons per s at 1.0 Å. To avoid radiation damage, the X-ray diffraction data were only collected at an oscillation angle of 20° per one protein crystal trapped into the microwell (total of 20 s exposure for each crystal). In case of the data set collection at 1.0 Å, the total absorbed dose calculated by RADDOSE Ver. 2 corresponds to 128 kGy.^40^ The protein crystals trapped into the microwells were sequentially measured and the collected diffraction data were automatically processed by KAMO.^41–43^ In the merging process, a hierarchical clustering method based on the unit-cell dimensions using the normalized structure factors was performed by XSCALE with outlier rejection protocol implemented in KAMO. For the crystals under cryo-conditions, complete diffraction datasets for each complex structure were collected using single crystals and each dataset was also processed by KAMO. The initial phases of the structure factors were solved by phenix.phaser using a search model (PDB code: 1RQW, 193L, and 1S0Q for thaumatin, lysozyme, and trypsin, respectively). Structure refinements were performed with the phenix program suite^44,45^ and COOT.^46^

## Results and discussion

### Sorting of the protein crystals into the microwells

The concept of our measurement system using a microfluidic protein crystal array device is illustrated in Fig. 1. Fig. 4 also shows a schematic illustration of the protein crystal sorting and the preparation process of the protein–ligand complex using the microfluidic device. Each microfluidic device consists of a microchannel layer and a microwell layer and the protein crystals were sorted by self-sedimentation and subsequently fixed to the microwells (Fig. 2 and 4c). Fixing into the microwells was based on two factors: (1) proteins can easily adsorb onto the PDMS surface by hydrophobic and electrostatic interactions and (2) the space limitation of the microchannel against the protein crystals affects crystal fixing. The protein crystals that were sorted into the microwells were continuously X-ray irradiated to collect the partial diffraction data at an oscillation angle of 20° per one crystal. Contrary to the conventional measurement method, the partial X-ray diffraction data from numerous protein crystals were merged to determine the 3D protein structure. For application of the microfluidic device to ligand screening, several solutions, including the protein crystal suspension, washing solution, and ligand solution, were introduced into the microchannel and microwells to wash the excess protein crystals and prepare the protein–ligand complexes, as shown in Fig. 4. Therefore, protein crystal should be fixed into the microwells to prevent the flow out of the crystals. The characteristics of the PDMS surface and the microchannel ensured that the protein crystals would be strictly fixed into the microwells to reduce the loss of protein crystals when replacing the solutions.

We next investigated the basis of our measurement concepts, namely sorting protein crystals into the microwells and replacing the solution in the microchannel. Movies S1 and S2† demonstrate the sorting step of the protein crystals into the microwells and the washing step in the microchannel, respectively. At the sorting step, we observed the sedimentation of the protein crystals in the depth direction, whereby the protein crystals were trapped into the microwells (Movie S1†). In addition, the sorted protein crystals were strictly fixed into the microwells and did not flow out from the microwells, even under high flow conditions (Movie S2†). On the other hand, the protein crystals that were sedimented onto the microchannel (not in the microwell) were easily removed during the washing step. The sorted protein crystals were continuously soaked with the ligands by introducing the ligand solution into the microchannel. Contrary to the traditional ligand soaking method, our method allows the simultaneous preparation of the protein–ligand complex simply by pipetting. This advantage is indispensable for X-ray diffraction measurement at RT, which requires tens of protein crystals to avoid any reduction in the X-ray diffraction intensity by radiation damage. Furthermore, numerous protein crystals were fixed into the periodically arrayed microwells. This allowed us to avoid the manual handling of the fragile protein crystals and the setting up of protein crystals onto the X-ray diffractometer.

Several types of platforms have been reported for serial X-ray diffraction measurement.^33,36^ However, in all the reported cases, the protein crystals were randomly placed into the platforms and thus, the position irradiated by X-ray was not determined in the platforms. Lyubimov *et al.* reported the application of a cell trap device^47^ for X-ray diffraction measurement.^33^ They demonstrated effective protein crystal capture using the cell trap device. However, the cell trap device required a syringe pump and stacking of the protein crystals was observed at the trap array. In contrast, our proposed microfluidic device only requires the use of a micropipette and the stacked protein crystals can be easily removed with the washing and ligand solutions. Thus, we considered the microwell-based platform suitable for on-site RT crystallography and application to ligand screening on the beamline at the synchrotron facility.

### Microwell size optimization for X-ray diffraction measurement

**Fig. 6:**
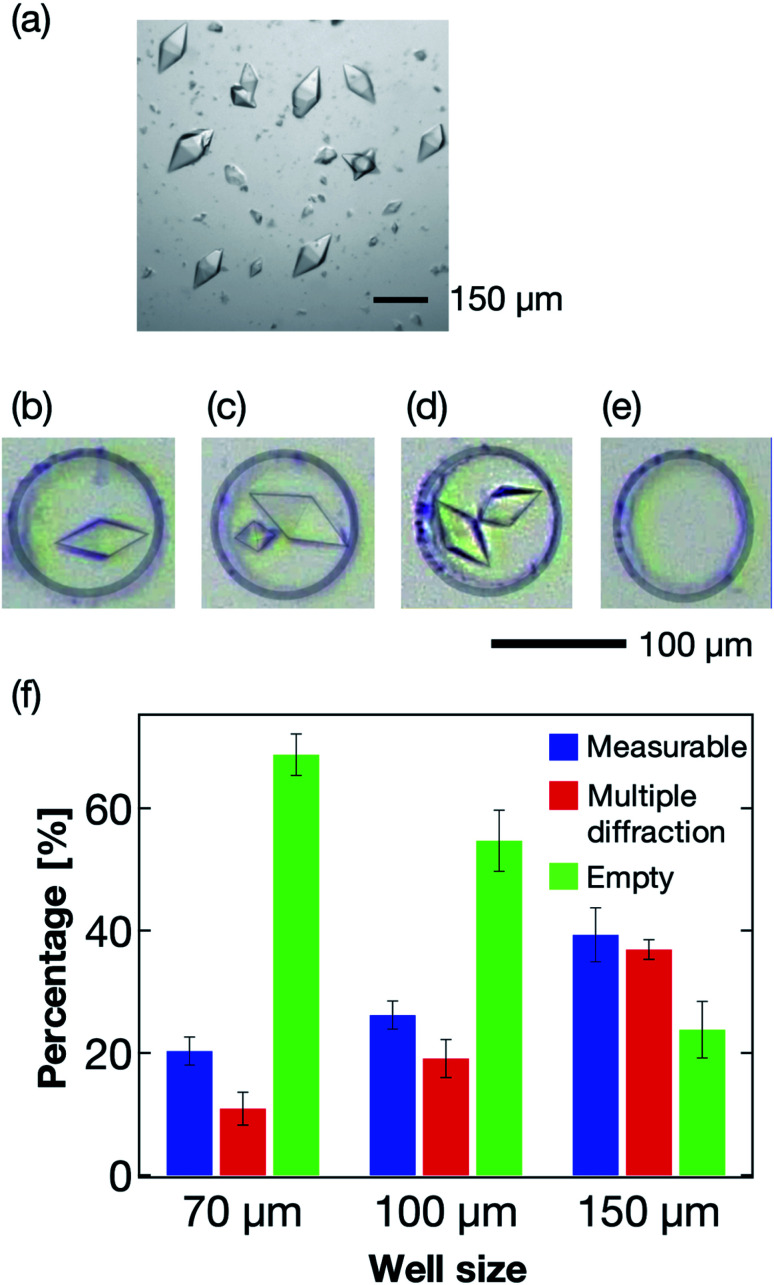
(a) Photograph of the thaumatin crystals used for the sorting experiments. The scale bar represents 150 μm. (b–e) Sorting patterns of the protein crystals in the microwell. The scale bar represents 100 μm. (b) Single sorting, (c) multiple sorting of crystals with a large size difference, (d) multiple sorting of crystals of similar size, and (e) empty microwell; microwell diameter = 100 μm. (f) Relationship between the microwell size and sorting patterns using thaumatin crystals. We counted 150–250 microwells for each microwell size. The error bars mean the standard deviation calculated from at least three reproducibility experiments. Definitions: measurable, percentage of the measurable microwells (b and c); multiple diffraction, percentage of the microwell sorted crystals of the same size (d); empty, percentage of empty microwells (e).

The microwell size is also a significant parameter to control the sorting of the protein crystals. Thus, we next optimized the size of the microwells to effectively sort the protein crystals into them. Typically, protein crystals exhibit a wide size distribution compared to those of cells and other micrometer-sized particles. For the thaumatin crystals, the size distributions used in this study were in the range 70–160 μm in the long axis direction (Fig. 6a). For the X-ray diffraction measurement, single sorting (one microwell containing one protein crystal; Fig. 6b) was established as the best condition out of the four tested sorting cases (Fig. 6b–e). When the average size of the protein crystals was smaller than the microwell size, the percentage of multiple sorting was predicted to increase (Fig. 6c and d). However, we confirmed that weak X-ray diffraction patterns from minor components could be eliminated at the analytical process if they exhibited large size differences. Therefore, we defined the single- and multi-sorted crystals with a double or large size difference as the measurable crystals (Fig. 6b and c). Fig. 6f illustrates the relationship between the microwell size and sorting patterns using thaumatin crystals. The sorting percentages were calculated from eqn (1):

1 Sorting rate = number of crystal sorted microwells/total number of microwells

Three types of microwells, 70, 100, and 150 μm in diameter, were employed for the sorting experiment. The results revealed the 150 μm microwell as the best of the three devices, with higher than 40% measurable crystals. We also confirmed that the percentage of empty microwells decreased with increasing microwell size. In contrast, the percentage of the multi-sorted microwells increased with increasing microwell size. These observations reveal that the proposed microfluidic device enables optimization of the microwell structure depending on the target protein crystal characteristics, namely the crystal size and shape. Thus, this measurement principle shows great potential as a versatile methodology for the X-ray protein structure analysis of a protein–ligand complex at RT.

### Proof of concept experiments using the microfluidic-based protein crystal array device

**Fig. 7:**
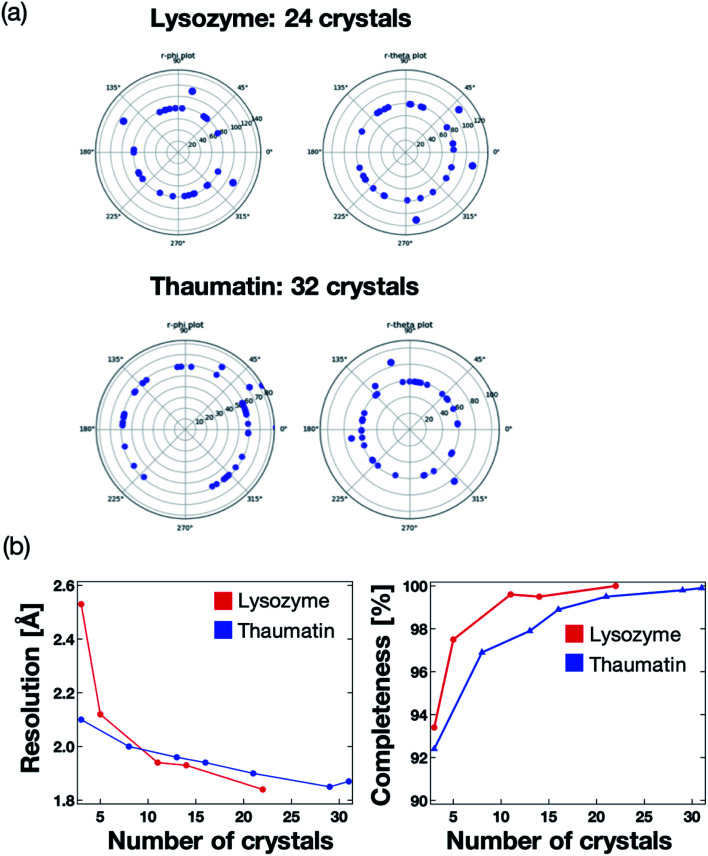
(a) Two-dimensional (2D) diagram of the crystal orientations. Each point displays the indexed crystals of lysozyme and thaumatin, with 24 and 32 measured crystals, respectively. The crystal orientations were referred from the UB matrix calculated by XDS software. Distances between the center of the circle and each blue point indicate an axis of the unit cell. Most crystals in the microfluidics were correctly indexed and oriented randomly. (b) Relationship between the crystallographic statistics (resolution and completeness) and number of measured crystals. The number of merged data was 22 and 31 for lysozyme and thaumatin, respectively.

We next validated the microfluidic-based measurement method for application to protein–ligand structure analysis. For the proof of concept experiment, thaumatin and lysozyme were measured using the microfluidic device on the BL26B2 beamline of SPring-8. A preferred orientation distribution of the protein crystals to the X-ray beam would also be significant for the collection of the complete X-ray diffraction data from a small angle range (20°) per one protein crystal. Fig. 7a illustrates the 2D diagram of the crystal orientations. The distances between the center of the circle and each blue point indicate the axis of the unit cell. Most crystals in the microfluidics were correctly indexed and oriented randomly. We measured 24 and 31 crystals for lysozyme and thaumatin, respectively. As a result, the protein crystals were randomly oriented to the X-ray beam. This result indicates that our method can determine the 3D protein structures by merging the diffraction data fixed into the microwells. The number of protein crystals necessary to determine the 3D structure depends on the space group.

The maximum resolution and data completeness were adopted as the benchmarks to evaluate the diffraction data. Based on the measurement principle and data in Fig. 7a, we concluded that the maximum resolution and data completeness could be improved by increasing the number of measured crystals. Fig. 7b presents the statistics of the X-ray diffraction data of lysozyme and thaumatin. Both the resolution and completeness were improved with increasing number of measured crystals. The lysozyme and thaumatin space groups are *P*4_3_2_1_2 and *P*4_1_2_1_2, respectively. Theoretically, the X-ray diffraction data at 90° per one crystal is required to collect the complete dataset, whereby the maximum resolution increases with increasing number of measured crystals. However, our method could determine the complete 3D lysozyme and thaumatin structures from at least three crystals randomly oriented to the X-ray beam. In addition, contrary to conventional protein crystallography, no manual crystal exchanges were required, giving our proposed system a strong advantage for RT protein–ligand complex structure analysis.

**Fig. 8:**
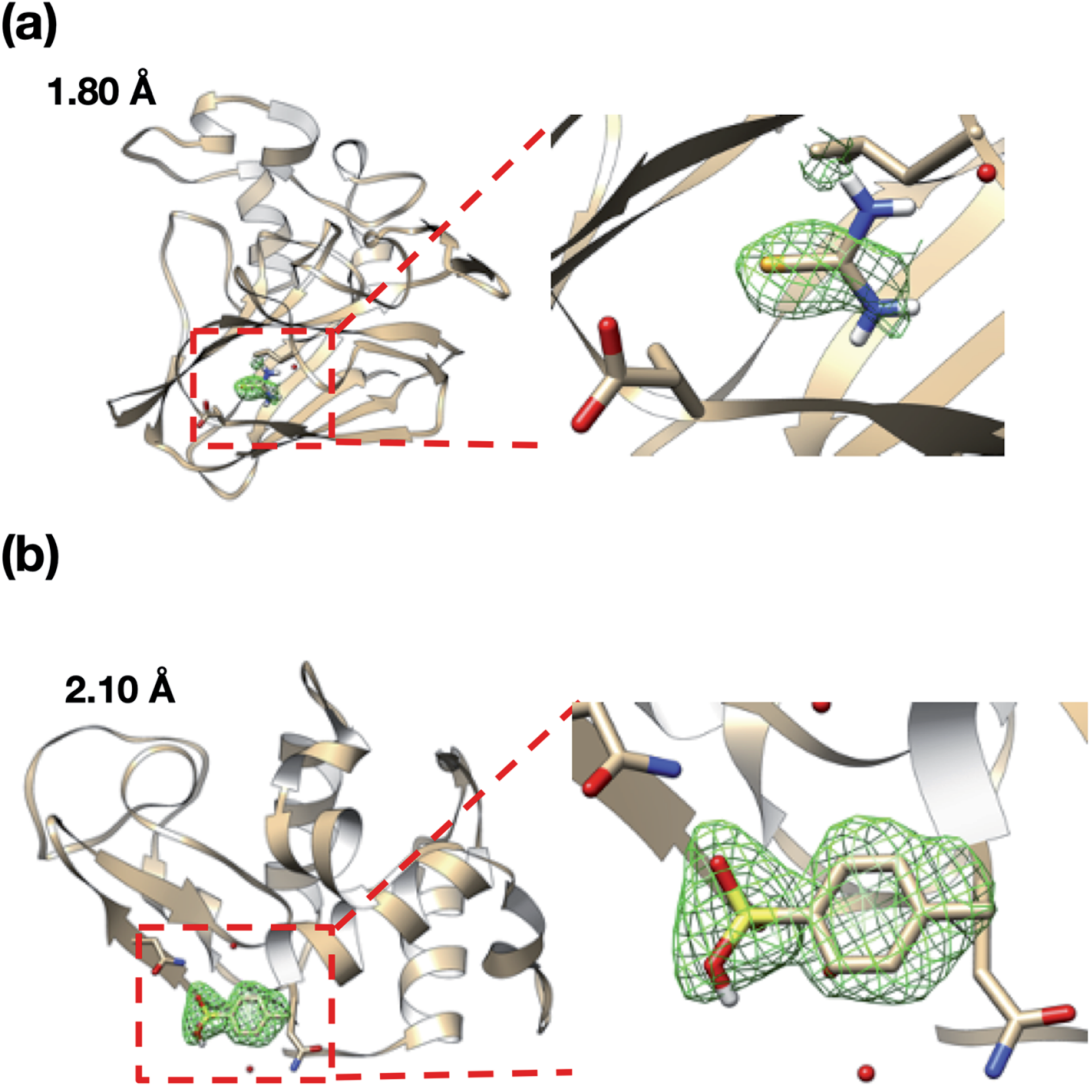
Omit maps for the (a) thaumatin–selenourea and (b) lysozyme–*p*-toluenesulfonic acid complexes. The mFo–DFc map was contoured at 2.0*σ*. Values on each figure represent the resolution of the outer shell.

We also attempted to measure the ligand complex of the thaumatin and lysozyme crystals as model proteins. Thus, after sorting the thaumatin/lysozyme crystals, the solution within the microfluidic device was replaced with the ligand solutions (selenourea/*p*-toluenesulfonic acid). Fig. 8 and Table 1 illustrate the omit maps and crystallographic data of the thaumatin–selenourea and lysozyme–*p*-toluenesulfonic acid complexes. At the binding sites, the differences in the electron densities of selenourea and *p*-toluenesulfonic acid were clearly observed from the merged X-ray diffraction data. Moreover, the crystallographic data statistics obtained from the numerous protein crystals were accurate enough to identify the binding sites of the ligands.

**Table 1:** Crystallographic statistics of lysozyme, the lysozyme–*p*-toluenesulfonic acid complex, thaumatin, and the thaumatin–selenourea complex. Values in brackets are for the highest resolution shell

Statistics	Lysozyme	Lysozyme complex	Thaumatin	Thaumatin-complex
Space group	*P*4_3_2_1_2	*P*4_3_2_1_2	*P*4_1_2_1_2	*P*4_1_2_1_2
Unit cell [Å, °]	*a* = *b* = 79.21	*a* = *b* = 79.12	*a* = *b* = 58.61	*a* = *b* = 58.96
	*c* = 38.26	*c* = 38.18	*c* = 151.80	*c* = 151.80
	*α* = *β* = *γ* = 90	*α* = *β* = *γ* = 90	*α* = *β* = *γ* = 90	*α* = *β* = *γ* = 90
Resolution limit [Å]	50–1.80 (1.91–1.80)	50–2.10 (2.23–2.10)	50–1.80 (1.91–1.80)	50–1.80 (1.91–1.80)
Redundancy	21.56 (21.10)	20.90 (21.01)	8.52 (8.59)	21.68 (21.78)
Completeness [%]	100 (100)	99.9 (99.8)	99.5 (99.9)	99.9 (100)
*R*_meas_ [%]	44.2 (271.8)	58.3 (287.6)	29.3 (157.7)	30.3 (170.9)
CC_1/2_ [%]	98.4 (73.5)	98.4 (69.9)	97.8 (62.1)	99.2 (81.1)
Measured crystals	24	28	13	32
Merged data	22	19	11	31

Coupling of the microfluidic-based protein crystal array device and automated diffraction image processing system (KAMO) improves the throughput of the protein–ligand complex 3D structure determination at RT. Moreover, RT crystallography leads to the better understanding of the physiological protein structure and functions. Thus, the features of our approach, namely RT crystallography using a protein crystal array device, provide many advantages for SBDD and FBDD, including ligand screening.

### Application to the ligand screening of trypsin

We next applied our method to the ligand screening of trypsin. This process required several microfluidic devices corresponding to the number of ligand compounds. Thus, we used thick-type microfluidic devices to evaluate the feasibility of mass production of the microfluidic device. We selected eight ligands for the ligand screening experiments as listed in Fig. 3. Melatonin (Fig. 3c) was selected as the negative control compound because it does not bind to trypsin. Benzamidine (Fig. 3e) was selected for this screening because the compound is a known trypsin inhibitor. The other ligands were selected based on their structural similarity to benzamidine; *i.e.*, their molecular weights and functional groups (most ligands have a basic functional group). In our knowledge, six structures other than benzamidine–trypsin complex and aniline–trypsin complex are novel complex structures.

**Fig. 9:**
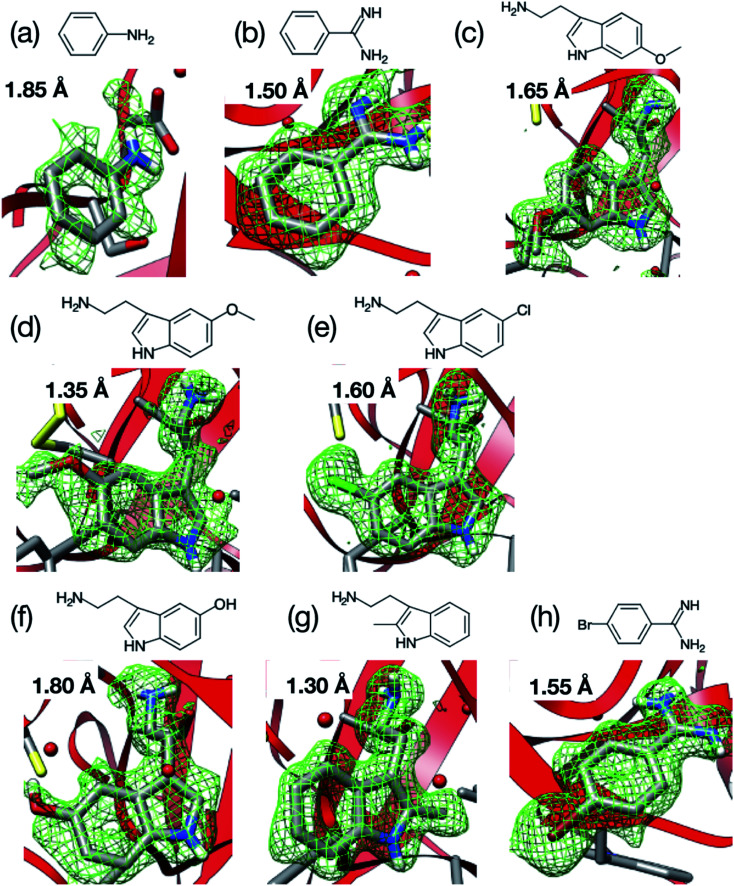
Room-temperature (RT) structures of the trypsin–ligand complex determined by the microfluidic device: (a) aniline (1.85 Å), (b) benzamidine (1.50 Å), (c) 6-methoxytryptamine (1.65 Å), (d) 5-methoxytryptamine (1.35 Å), (e) 5-chlorotryptamine (1.60 Å), (f) serotonin (1.80 Å), (g) 2-methyltryptamine (1.30 Å), and (h) 4-bromobenzamine (1.55 Å). Values on each figure and inside the parentheses represent the resolution of the outer shell. The mFo–DFc map was contoured at 2.0*σ*. Ligand screening of trypsin was performed using nine ligands and the electron densities of eight out of nine ligands were observed in the binding site of trypsin.

For comparison, diffraction data from cryo-cooled samples of all the tested complexes were also collected. All the X-ray diffraction experiments using the microfluidic device could cover sufficient data completeness. Aside from melatonin, the electron density corresponding to each ligand was clearly observed at the ligand binding site of trypsin (Fig. 9). These results indicate that the thick-type microfluidic device also makes the use of RT crystallography and its application to ligand screening possible. The microfluidic device was fabricated by a rapid prototyping process and used disposable. This is a major advantage for the ligand screening to prevent cross-contamination. Notably, the high-resolution limits of the datasets from the proposed microfluidic device were lower than those afforded under cryo-conditions (Table 2). This was mainly attributed to the thermal fluctuations of the protein molecules in the RT crystals being higher than those of the cryogenic crystals. As expected, the Wilson B-factors of the datasets and averaged B-factors of the structures at RT tended to be higher than those observed under cryogenic conditions, and the occupancies of the compounds for both conditions were almost the same (Table 2). This indicates that the structural analysis of the protein–ligand binding interactions at RT potentially provides results comparable to those attained by cryogenic crystallography.

Data collection and refinement statistics under (top) room-temperature (RT) and (bottom) cryogenic conditions

**Table:** 

Room temperature								
	Aniline	Serotonin	2-Methyltryptamine	4-Bromobenzamidine	5-Chlorotryptamine	5-Methoxytryptamine	6-Methoxytryptamine	Benzamidine
PDB ID	7BS0	7BS2	7BRZ	7BRV	7BRW	7BRX	7BRY	7BS1

**Data collection**								
Space group	*P*2_1_2_1_2_1_	*P*2_1_2_1_2_1_	*P*2_1_2_1_2_1_	*P*2_1_2_1_2_1_	*P*2_1_2_1_2_1_	*P*2_1_2_1_2_1_	*P*2_1_2_1_2_1_	*P*2_1_2_1_2_1_

**Cell dimensions**								
*a*, *b*, *c* (Å)	55.18, 59.31, 67.69	55.09, 58.52, 67.58	54.92, 58.59, 67.64	54.98, 58.77, 68.03	55.22, 58.92, 67.75	54.90, 58.60, 67.44	55.33, 57.21, 67.42	54.45, 57.88, 66.69
*α*, *β*, *γ* (°)	90.00, 90.00, 90.00	90.00, 90.00, 90.00	90.00, 90.00, 90.00	90.00, 90.00, 90.00	90.00, 90.00, 90.00	90.00, 90.00, 90.00	90.00, 90.00, 90.00	90.00, 90.00, 90.00
Resolution (Å)	44.61–1.85 (1.92–1.85)	44.24–1.80 (1.86–1.80)	33.82–1.30 (1.35–1.30)	40.15–1.55 (1.61–1.55)	44.46–1.6 (1.66–1.6)	34.44–1.35 (1.40–1.35)	39.77–1.65 (1.71–1.65)	34.09–1.50 (1.55–1.50)
*R*_meas_	0.296 (1.562)	0.207 (0.794)	0.147 (3.344)	0.185 (2.268)	0.289 (1.919)	0.154 (4.236)	0.152 (1.387)	0.128 (2.916)
〈*I*/*σ*(*I*)〉	4.52 (0.94)	7.21 (2.26)	15.47 (2.05)	10.13 (1.71)	4.91 (1.00)	21.42 (3.28)	9.42 (1.33)	15.74 (0.93)
CC_1/2_	0.968 (0.316)	0.979 (0.683)	0.999 (0.406)	0.998 (0.590)	0.967 (0.337)	0.999 (0.463)	0.994 (0.487)	0.995 (0.302)
Completeness (%)	94.55 (95.05)	92.61 (91.34)	99.67 (99.59)	99.10 (99.15)	96.01 (95.06)	99.02 (99.62)	95.60 (97.30)	92.00 (84.09)
Redundancy	3.6 (3.4)	3.7 (3.7)	9.3 (8.6)	10.3 (10.0)	5.4 (5.3)	12.8 (12.0)	6.6 (6.5)	15.7 (13.8)
Wilson B-factor	15.99	11.68	11.31	13.99	13.53	12.66	15.41	15.66

**Refinement**								
No. unique reflections	18 476 (1804)	19 327 (1877)	54 271 (5306)	32 276 (3162)	28 648 (2791)	48 000 (4761)	25 238 (2523)	31 682 (2864)
*R*_*w*ork_/*R*_free_	0.1769/0.2107	0.1491/0.1936	0.1621/0.1837	0.1645/0.1759	0.1728/0.2143	0.1630/0.1815	0.1598/0.1961	0.1688/0.1898

**No. atoms**								
Protein	1682	1674	1701	1711	1746	1759	1675	1654
Ligand	8	19	19	25	14	15	20	10
Water	202	222	205	140	208	197	174	199

**Averaged B-factors (Å** ^**2**^ **)**								
Protein	18.52	13.82	14.37	16.99	16.64	15.85	17.84	16.52
Ligand	24.45	25.23	20.57	32.83	18.34	25.31	28.31	16.29
Water	31.26	30.07	29.34	29.39	31.71	30.32	31.41	29.65

**R.m.s. deviations from ideal**								
Bond lengths (Å^2^)	0.007	0.01	0.005	0.006	0.007	0.01	0.005	0.01
Bond angles (°)	0.79	0.77	0.78	0.82	0.80	1.30	0.79	0.84

**Ramachandran plot**								
Favored (%)	98.64	97.74	98.64	98.19	97.74	97.74	98.19	98.19
Allowed (%)	1.36	2.26	1.36	1.81	2.26	2.26	1.81	1.81
Outlier (%)	0.00	0.00	0.00	0.00	0.00	0.00	0.00	0.00
Ligand occupancy	0.64	0.86	0.90	0.69	0.81	0.67	0.84	0.93

**Table:** 

Cryogenic-conditions								
	Aniline	Serotonin	2-Methyltryptamine	4-Bromobenzamidine	5-Chlorotryptamine	5-Methoxytryptamine	6-Methoxytryptamine	Benzamidine
PDB ID	7BS7	7BS9	7BS6	7BS3	7BSA	7BS4	7BS5	7BS8
Data collection								
Space group	*P*2_1_2_1_2_1_	*P*2_1_2_1_2_1_	*P*2_1_2_1_2_1_	*P*2_1_2_1_2_1_	*P*2_1_2_1_2_1_	*P*2_1_2_1_2_1_	*P*2_1_2_1_2_1_	*P*2_1_2_1_2_1_

**Cell dimensions**								
*a*, *b*, *c* (Å)	54.71, 58.57, 66.83	54.74, 58.53, 66.62	54.61, 58.56, 66.86	54.24, 56.90, 66.67	54.38, 58.57, 66.66	54.56, 58.67, 66.50	54.84, 56.90, 66.57	54.11, 57.07, 66.17
*α*, *β*, *γ* (°)	90.00, 90.00, 90.00	90.00, 90.00, 90.00	90.00, 90.00, 90.00	90.00, 90.00, 90.00	90.00, 90.00, 90.00	90.00, 90.00, 90.00	90.00, 90.00, 90.00	90.00, 90.00, 90.00
Resolution (Å)	33.42–1.04 (1.08–1.04)	39.98–1.05 (1.09–1.05)	33.43–1.04 (1.08–1.04)	42.28–1.28 (1.33–1.28)	39.85–1.12 (1.16–1.12)	26.84–1.04 (1.08–1.04)	39.49–1.17 (1.21–1.17)	33.77–1.37 (1.43–1.37)
*R*_meas_	0.063 (0.667)	0.053 (0.549)	0.039 (0.144)	0.127 (1.664)	0.092 (1.128)	0.032 (0.477)	0.081 (1.391)	0.040 (1.026)
〈*I*/*σ*(*I*)〉	24.90 (1.81)	28.55 (2.49)	48.71 (8.12)	12.06 (1.26)	21.24 (1.86)	43.93 (2.70)	21.31 (1.65)	42.91 (2.90)
CC_1/2_	0.999 (0.738)	0.999 (0.821)	0.999 (0.984)	0.999 (0.476)	1.000 (0.614)	1.000 (0.835)	1.000 (0.627)	1.000 (0.906)
Completeness (%)	88.20 (28.65)	87.46 (34.66)	87.55 (26.88)	99.91 (99.23)	92.70 (60.46)	88.00 (26.66)	99.25 (92.70)	98.99 (96.96)
Redundancy	12.1 (3.8)	12.5 (4.5)	12.1 (3.7)	7.6 (7.4)	21.24 (1.86)	11.9 (3.7)	13.8 (9.8)	14.4 (14.4)
Wilson B-factor	8.41	9.36	6.34	10.54	8.63	8.64	10.65	16.22

**Refinement**								
No. unique reflections	90 800 (2916)	87 417 (3404)	90 512 (2748)	102 008 (10 148)	76 437 (4930)	90 569 (2714)	70 079 (6479)	42 915 (4142)
*R*_work_/*R*_free_	0.1583/0.1690	0.1540/0.1703	0.1518/0.1619	0.1747/0.1907	0.1575/0.1752	0.1540/0.1718	0.1668/0.1807	0.1616/0.1781
No. atoms								
Protein	1673	1669	1658	1681	1656	1703	1681	1696
Ligand	34	35	26	59	57	77	27	23
Water	383	442	394	242	331	416	360	307

**Averaged B-factors (Å** ^**2**^ **)**								
Protein	11.96	10.50	7.50	12.53	10.63	12.99	12.66	16.89
Ligand	9.82	14.96	10.45	23.14	14.70	10.32	15.68	22.08
Water	17.82	22.09	18.56	25.39	22.91	16.78	25.8	30.12

**R.m.s. deviations from ideal**								
Bond lengths (Å^2^)	0.004	0.004	0.005	0.01	0.005	0.01	0.005	0.01
Bond angles (°)	0.84	0.83	0.88	0.88	0.85	0.86	0.82	0.85

**Ramachandran plot**								
Favored (%)	98.64	98.64	97.74	98.19	98.64	98.19	97.74	98.64
Allowed (%)	1.36	1.36	2.26	1.81	1.36	1.81	2.26	1.36
Outlier (%)	0.00	0.00	0.00	0.00	0.00	0.00	0.00	0.00
Ligand occupancy	0.61	0.78	0.88	0.70	0.86	0.70	0.85	0.86

**Fig. 10:**
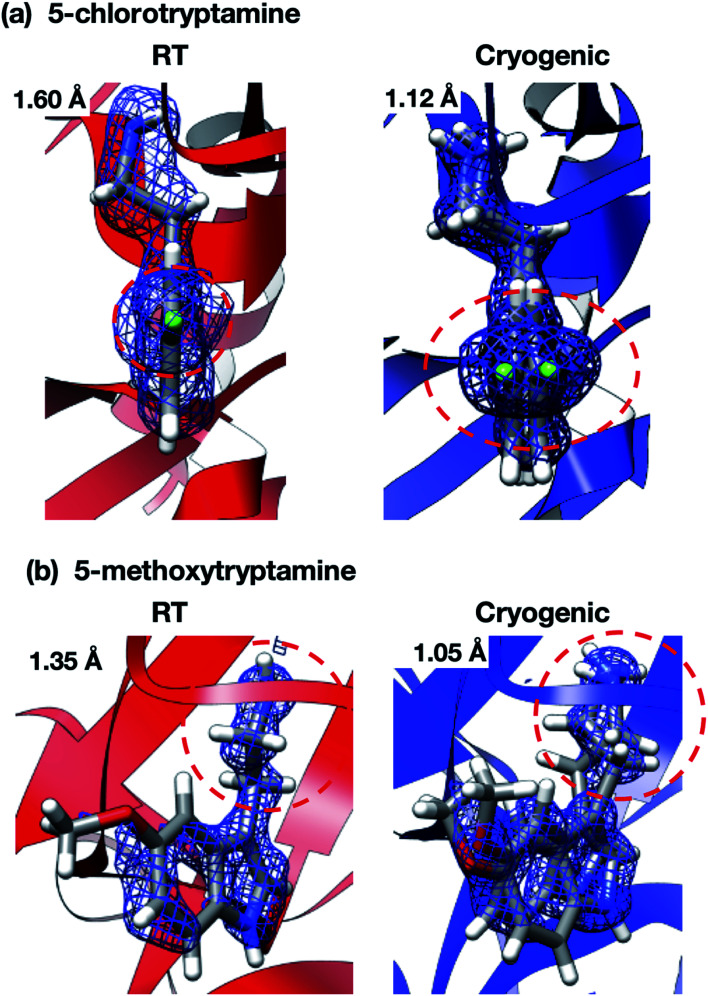
Alternate conformations of (a) 5-chlorotryptamine and (b) 5-methoxytryptamine were only observed in the cryogenic structures. The 2mFo–DFc map of each ligand was contoured at 1.0*σ*. Red dashed circles represent the main alternate conformation moiety of the ligands. Clear alternate conformations were only observed in the cryogenic structures (red circles).

**Fig. 11:**
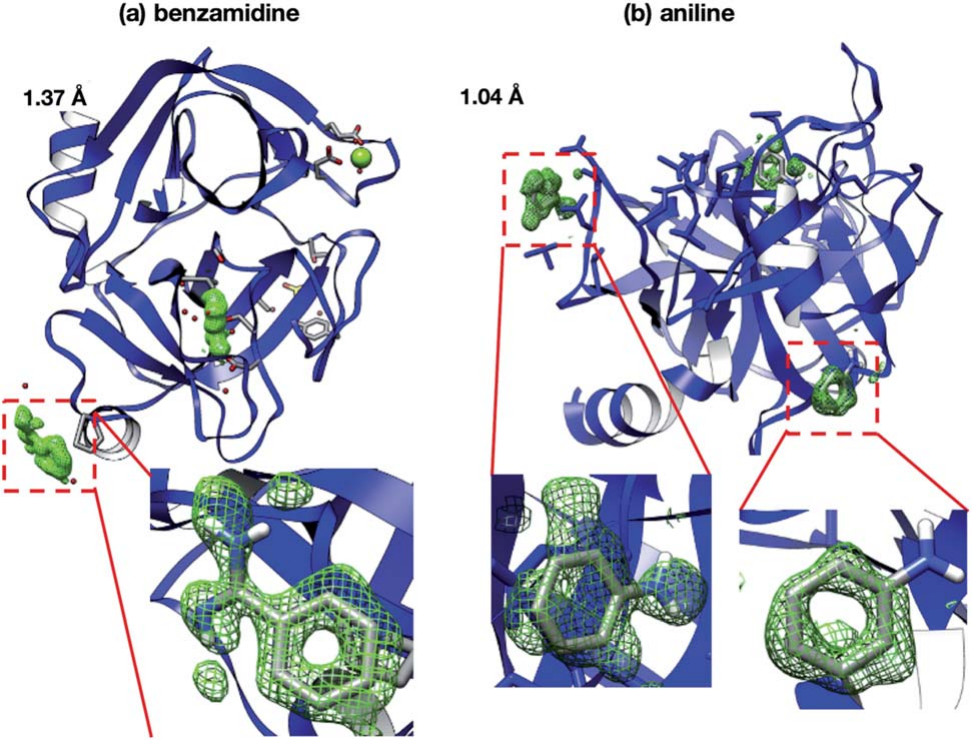
Extra binding sites in (a) benzamidine and (b) aniline observed only under cryo-conditions. Electron density maps from benzamidine and aniline were observed (mFo–DFc map contoured at 2.0*σ*). These binding sites are physiologically meaningless because trypsin does not have binding regions in these sites.

Interestingly, the structures of 5-chlorotryptamine and 5-methoxytryptamine attained under RT and cryogenic conditions were slightly different. Thus, the two ligands adopted alternate conformations only under cryo-conditions (Fig. 10), possibly due to the presence of a cryoartifact, *i.e.* an external force on the flash cooling. Furthermore, for both benzamidine and aniline, additional ligand-bound sites were only observed under cryogenic conditions (Fig. 11). It is well known that trypsin has a single binding site^48^ located near Asp194, and all the binding sites of the additional ligands observed in benzamidine and aniline were located at the surface of the protein molecules. These results indicate that the extra ligands obtained under cryo-conditions in this study are physiologically meaningless. These binding sites are also considered to be artifacts caused by freezing, and such non-physiological ligand binding is undesirable in the ligand optimization process for FBDD. This implies that *in situ* crystallography might have an advantage in ligand screening, especially when the binding affinity of a ligand is weak.

The collective results reveal that compared to the methodology currently in use, ligand screening using the proposed microfluidic-based protein crystal array device can provide more natural structures at ease, while drug development by FBDD could be more effective.

## Conclusion

In this study, we developed microfluidic devices named “protein crystal array devices” for RT protein crystallography applicable for protein–ligand complex 3D structure analysis. We confirmed the concept of the microfluidic sorting of protein crystals into microwells. Owing to the strong fixing of the protein crystals to the microwells, the protein crystals could be spontaneously sorted solely by a pipetting procedure and were subsequently measured at RT. The collected X-ray diffraction data from the multiple protein crystals were automatically processed and merged to determine the 3D protein structure. Notably, our approach did not require any external apparatus such as syringe pumps and skilled handling for protein crystallography and microfluidics.

The protein crystal array device allowed the simultaneous preparation of multiple protein–ligand complex crystals by replacing the microchannel content with each ligand solution. We determined eight trypsin–ligand complex structures at RT and found differences in the configurations of two compounds, compared to the cryogenic counterparts, as well as extra binding sites to trypsin in the cryogenic structures. Therefore, we supposed that the protein–ligand complex analysis based on RT protein crystallography might allow a more detailed understanding of protein–ligand interactions, even for interactions with weak affinities. Owing to these features, RT crystallography using our microfluidic device shows great potential for application to ligand screening for SBDD and FBDD. In the present study, we carried out RT crystallography using the microfluidic device manually to demonstrate the simplicity of our approach. However, the microfluidic device can also be integrated to multi-microchannels and -microwells for high-throughput ligand screening to accelerate the automation of RT crystallography and ligand screening. The advantage of our device which allows simple and easy handling of samples would be a great benefit for developing an automated system of sample preparation. We believe that the proposed protein–ligand complex structure analysis and its application to ligand screening at RT can provide significant information for the better understanding of protein function under physiological conditions and the development of new drugs.

## Author contributions

Conceptualization: M. M., S. I., and G. U.; data curation: R. T., S. I., M. M., and G. U.; formal analysis: S. I., R. T., M. M, G. U., A. I., H. T., M. Y., and M. T.; methodology: M. M., S. I., and G. U.; investigation: R. T., S. I., M. M., and G. U.; writing of the original draft: M. M. and S. I.; writing review and editing: M. M., S. I., G. U., and M. T.; funding acquisition: M. M., G. U., and Y. M.; resources: R. T., S. I., M. M. and G. U.; supervision: M. M. and Y. M.

## Conflicts of interest

There are no conflicts to declare.

## Supplementary Material

SC-011-D0SC02117B-s001

SC-011-D0SC02117B-s002
